# Novel mobility index tracks COVID-19 transmission following stay-at-home orders

**DOI:** 10.1038/s41598-022-10941-2

**Published:** 2022-05-10

**Authors:** Peter Hyunwuk Her, Sahar Saeed, Khai Hoan Tram, Sahir R Bhatnagar

**Affiliations:** 1 Department of Pharmacology and Therapeutics, McGill University, Montreal, Canada; 2 Department of Medical Biophysics, University of Toronto, Toronto, Canada; 3 Division of Infectious Diseases, Department of Medicine, Washington University School of Medicine, St. Louis, USA; 4 Department of Public Health Sciences, Queen’s University, Ontario, Canada; 5 Division of Infectious Diseases, Department of Medicine, University of Washington, Seattle, USA; 6 Department of Epidemiology, Biostatistics and Occupational Health, McGill University, Montreal, Canada; 7 Department of Diagnostic Radiology, McGill University, Montreal, Canada

**Keywords:** Epidemiology, Computational models, Software, Statistical methods

## Abstract

Considering the emergence of SARS-CoV-2 variants and low vaccine access and uptake, minimizing human interactions remains an effective strategy to mitigate the spread of SARS-CoV-2. Using a functional principal component analysis, we created a multidimensional mobility index (MI) using six metrics compiled by SafeGraph from all counties in Illinois, Ohio, Michigan and Indiana between January 1 to December 8, 2020. Changes in mobility were defined as a time-updated 7-day rolling average. Associations between our MI and COVID-19 cases were estimated using a quasi-Poisson hierarchical generalized additive model adjusted for population density and the COVID-19 Community Vulnerability Index. Individual mobility metrics varied significantly by counties and by calendar time. More than 50% of the variability in the data was explained by the first principal component by each state, indicating good dimension reduction. While an individual metric of mobility was not associated with surges of COVID-19, our MI was independently associated with COVID-19 cases in all four states given varying time-lags. Following the expiration of stay-at-home orders, a single metric of mobility was not sensitive enough to capture the complexity of human interactions. Monitoring mobility can be an important public health tool, however, it should be modelled as a multidimensional construct.

## Introduction

While highly effective vaccines are readily available in the United States, uptake remains low^1,2^ and interventions aimed at minimizing human contact continue to be necessary to mitigate the spread of SARS-CoV-2^3–8^. The potential of monitoring population-level mobility patterns using geolocated mobile phone data as a public health tool has been demonstrated^9–12^. In March 2020, worldwide adherence to lockdowns was measured using various mobility metrics^13–18^. A modelling study from China showed 20–60% reductions in mobility notably controlled the spread of SARS-CoV-2^19^. A study from Canada showed that reductions in mobility strongly predicted future control of SARS-CoV-2 growth rates^6^. However, in the absence of social distancing interventions, the association between changes in population-level mobility and COVID-19 remains unclear^11,12^. This is particularly important now as highly transmissible variants of concerns (Delta, Omicron) have become dominant strains^20^ and transmission is likely to occur before clinical cases are diagnosed^21^. Additionally, relying on case detection alone to predict surges in transmission continues to underestimate the pandemic as molecular diagnostic tests have become scarce worldwide^22^. Thus, the need to rapidly identify populations and locations at heightened risk of exposure remains necessary.

Population-level mobility, as it pertains to human interaction, is multidimensional. This is particularly true when assessing distinct geographical areas that vary by population density and socioeconomic factors across the United States^23,24^. While the measurement of mobility is complex, most studies to date have used single metrics such as the percentage of people remaining at home or changes in the distance travelled to summarize human interactions and evaluate trends and associations with COVID-19^9^. These single metrics may oversimplify mobility associated with human interaction. As social distancing policies loosened from strict “lock down” to business-as-normal, the utility of continuously monitoring mobility may require a robust definition that is able to capture the complexity of population-level movement^25^. To this end, the aim of this study was to use advanced statistical methods to create a novel index that summarizes mobility as a latent construct using a combination of six mobility metrics. We evaluated how our mobility index varied across 365 counties in 4 states as a function of time. Finally, we assessed the validity of our mobility index by evaluating how mobility correlated with COVID-19 cases from four states in the Midwest compared to a single metric from the time stay-at-home orders expired.

## Methods

### Data sources

#### Mobility metrics

We used aggregated mobility data publicly available through SafeGraph from January 1 to December 8, 2020, via the Social Distancing Metric database. SafeGraph uses a panel of GPS pings from anonymous mobile devices from a representative sample of the US Census population to derive metrics of mobility. These data includes a range of spatial behaviors from >45 million mobile devices ($\approx$ 10% of devices in the United States). To enhance privacy, SafeGraph excludes census block group information if fewer than two devices visited an establishment in a month.

*A priori*, we choose six mobility metrics commonly used in the literature as a proxy of human contact and that could be attributable to mobility behavior changes as associated with COVID-19 infections. Each metric is defined for a given day (t) for a given county (j). The metrics (s) included:

- The fraction of devices leaving home in a day
- The fraction of devices away from home for 3–6 hours (Part-time work behavior)
- The fraction of devices away from home longer than 6 hours (Full-time work behavior)
- The median time spent away from home
- The median distance traveled from home
- The average number of short stops (>3 stops for less than 20 min) (Delivery behaviors).

#### COVID-19 cases

Confirmed COVID-19 cases data were retrieved from the New York Times open-source project^26^. This publicly available dataset aggregates county-level daily counts of diagnosed cases, from Health services’ official reports. All methods were carried out in accordance with the relevant guidelines.

#### Covariates

Demographic variables including population size and population density for each county were collected from the American Community Survey, data available through the the US Census Bureau. To capture the variability of health, social, and economic factors across counties we used the COVID-19 Community Vulnerability Index (CCVI)^27,28^. The CCVI is similar to the Social Vulnerability Index, which was developed by the CDC to support disaster management^29^ except with additional elements specific to the COVID-19 pandemic. The index combines 40 indicators of vulnerability from seven themes (socioeconomic status, minority status, household composition, epidemiology, healthcare system, high risk environments, population density) to create the county and state-level index. The CCVI is recognized by the Centers for Disease Control and Prevention as a valuable tool in COVID-19 research and pandemic response planning^30,31^. Dates for when stay-at-home orders were enforced and lifted were obtained from the state-level social distancing policies database^32^. There were several entries for each state due to policy revisions or updates. We used the first entry for each state in this database.

### Analysis

#### Population

Given the magnitude of the available data, we reduced the number of states in our analysis to four populous and neighboring states in the Midwest. These states had similar numbers of counties and socioeconomic indicators. The counties from each state represented varying population densities and rural vs. urban areas. Based on the 2020 Presidential elections, Illinois (IL) and Michigan (M) were considered Democratic states while Ohio (OH) and Indiana (IN) were predominately Republican states. We used every county from each of the selected states to avoid any preferential selection.

#### Mobility index

We defined mobility as a change of each mobility metric relative to the average of the week before (time-updated rolling average). For each county $j=1, \ldots , 365$, we index each of the 6 mobility metrics $s = 1, \ldots , 6$ by calendar day $t = 1, \ldots , m_j$, where $m_j$ is the total number of observed days since re-opening in county *j*. We define the following quantities:

- $X_{j,t,s}$: the scalar value of mobility metric *s* measured on day *t* in county *j*.
- ${\varvec{{X}}}_{j,t-8,\ldots ,t-1, s} = (X_{j,t-8,s}, \ldots , X_{j,t-1,s}) \in \mathbb {R}^8$: the value of mobility metric *s* measured on days $t-8, \ldots , t-1$, i.e., the 7 days prior to day *t* in county *j*. This is a vector quantity.
- $\overline{X}_{j,t-8,\ldots ,t-1,s}$: the average of the ${\varvec{{X}}}_{j,t-8,\ldots ,t-1,s}$.

The change from baseline mobility metric *s* for day *t* in county *j* is given by

1 $$\begin{aligned} \Delta X_{j,t,s} = \frac{X_{j,t,s} - \overline{X}_{j,t-8,\ldots ,t-1,s}}{{\overline{X}_{j,t-8,\ldots ,t-1,s}}} \end{aligned}$$

The use of a rolling average is unique to this analysis. Most studies have used a static relative baseline period such as mobility trends between January until February 2020^9,33,34^. This common approach does not account for seasonal mobility variability or changes as a result of the pandemic^35–37^. In contrast, our baseline (rolling average) takes into consideration temporal trends that were likely changing with evolving public health policies. To check for outliers and appropriateness of the use of the average, we calculated the coefficient of variation (CV) for each individual metric in each county (Supplemental Figure S13). We found that there were no strong outliers, as all the CV were less than 2.5, suggesting that the average was a valid metric to use. We also considered using the median but did not find a significant difference in the results relative to the average.

Since our hypothesis was each metric could be attributed to a common underlying notion of mobility, we used an unsupervised machine learning method known as functional principal component analysis (fPCA) to create our latent mobility index^38^. Briefly, PCA is a technique for reducing the dimensionality of multiple variables while minimizing information loss. This is done by creating new uncorrelated variables (principal components) that successively maximize variance. A “functional” PCA accounts for the longitudinal nature of the data. We applied fPCA on $\Delta X_{j,t,s}$ separately for each county and extracted the first principal component, i.e., the linear combination of individual mobility metrics that explained the most variance. We denote this first principal component by fPCA$_{j,t}$, a score summarizing mobility in each county (*j*) on a given day (*t*). To enable comparability between counties and states, fPCA$_{j,t}$ were scaled as Z-scores, which defined our mobility index (MI) given by:

2 $$\begin{aligned} MI_{j,t} = \frac{fPCA_{j,t} - \overline{fPCA}_{j,t}}{\sqrt{Var(fPCA_{j,t})}}, \end{aligned}$$

where $\overline{fPCA}_{j,t} = \frac{1}{m_j}\sum _{k=1}^{m_j} fPCA_{j,k}$ and $Var(fPCA_{j,t})$ are the average and variance of the fPCA scores in county *j* over the observed time period, respectively.

The interpretation of *MI* is as follows: $MI=0$, on average there was no change in mobility relative the previous week; $MI=1$ on average there was an increase in mobility by one standard deviation relative to the last week and: $MI=-1$ on average there was a decrease in mobility by one standard deviation relative to the last week. An animation was created to visualize the relative daily changes of *MI* by counties.

#### Association with COVID-19

For each county *j*, let $y_{j,t}$ be the number of confirmed COVID-19 cases on day $t = 1, \ldots , m_j$, and $\mathbf {q}_{j,t} = [MI_{j,t-0}, \ldots , MI_{j,t-21}]$ denote the vector of lagged occurrences of our mobility index (defined in Eq. (2)) with 0 days and 21 days as minimum and maximum lags, respectively. In words, the first element of $\mathbf {q}_{j,t}$ represents the value of our mobility index on day *t*, the second element represents the value of our mobility index one day prior to *t*, and so on. From the time stay-at- home orders expired until December 8 2020, the relationship between daily counts of COVID-19 cases ($y_{j,t}$) and mobility ($\mathbf {q}_{j,t}$), accounting for up to 21 days of lag, was estimated with a quasi-Poisson hierarchical generalized additive model (HGAM)^39,40^ of the form:

3 $$\begin{aligned} \log (E(y_{j,t}))&= \beta _0 + s(\mathbf {q}_{j,t}) + s(\text {{time}}_t) + s_j(\text {{time}}_t) + CCVI_{j} + density_j, \end{aligned}$$

where $\beta _0$ is the intercept, $s(\cdot )$ are the smooth non-parametric functions of the predictor variables, $CCVI_j$ is the COVID-19 Community Vulnerability Index and $density_j$ is the population density (people per square kilometer) at the county-level. The term $s(\mathbf {q}_{j,t})$ in Eq. (3) captures the potentially non-linear and delayed effect of mobility on COVID-19 cases through a cross-basis function^41^. We used penalized cubic regression splines^39^ for both dimensions, with interior knots placed at Z-scores of $-3, -2,-1,0,1,2,3$ for $MI_{j,t}$, and 7 and 14 days for the lag. The penalty is given by $\lambda \varvec{\beta }^\top \mathbf {S}\varvec{\beta }$, where $\varvec{\beta }$ are the regression parameters, $\mathbf {S}$ is a matrix of known coefficients^42^, and $\lambda$ is the tuning parameter that controls the degree of smoothing and chosen via generalized cross-validation. Given the heterogeneity of COVID-19 epidemiology across counties, models included both a state level calendar time effect $s(\text {{time}}_t)$ using thin plate regression splines^43^ and a county level calendar time effect $s_j(\text {{time}}_t)$ using a factor-smoother interaction basis^40^. Population size was used as offset in each model. A separate model was run for each of the selected states.

There were two main advantages for using a HGAM to evaluate the association between mobility and COVID-19 cases: (1) it can quantify the non-linear functional relationships over time where the shape of each function varies across counties, and (2) it has the capacity of modelling varying lags^44^. Based on a recent systematic review of 42 studies, the mean incubation period of SARS-CoV-2 was 8 days (95% CI 10.3, 16 days)^45^. This lag between time of infection and becoming symptomatic/testing positive can vary at both the patient-level (the time it takes to develop symptoms and get tested) and also at the county-level (the time it takes to perform and report the test results). Given this variation, we were able to control for varying lagged exposures (up to 21 days) at the county-level. To evaluate the utility of our mobility index, we compared a dose-response relationship between mobility and COVID-19 cases and goodness-of-fit-statistics of our latent MI compared to a single measure of mobility (the fractions of devices leaving the home). All analyses were performed using R version 4.0.2^46^ along with the mgcv^39^ and dlnm^47^ packages. Code and data for reproducing all the results, figures and animation in this paper is available at https://github.com/sahirbhatnagar/covid19-mobility.

## Results

### Mobility patterns

Daily mobility changes of three hundred sixty-five counties from the four most populous states in the Midwest: Illinois, Ohio, Michigan and Indiana were analyzed between January 1 2020 until December 8 2020. State-level sociodemographic and economic characteristics were similar across four states and are summarized in Table 1.

**Table 1:** The sociodemographic and economic characteristics of Illinois (IL), Ohio (OH), Michigan (M) and Indiana (IN).

State	Order	Lift	Population	Number of counties	Median household income	Cumulative cases per capita at opening$$^{a}$$	Cumulative cases per capita until Dec 8$$^{a}$$	Party affiliation$$^{b}$$
Illinois	March 21	April 8	12,741,080	102	$65,030	118	6324	Democratic
Ohio	March 24	April 7	11,689,442	88	$56,111	41	4363	Republican
Michigan	March 24	April 14	9,995,915	83	$56,697	269	4427	Democratic
Indiana	March 25	April 7	6,691,878	92	$55,746	83	5909	Republican

$^\mathrm{a}$Cumulative cases per 100,000 population

$^\mathrm{b}$Based on the 2020 Presidential elections^48^.

**Figure 1:**
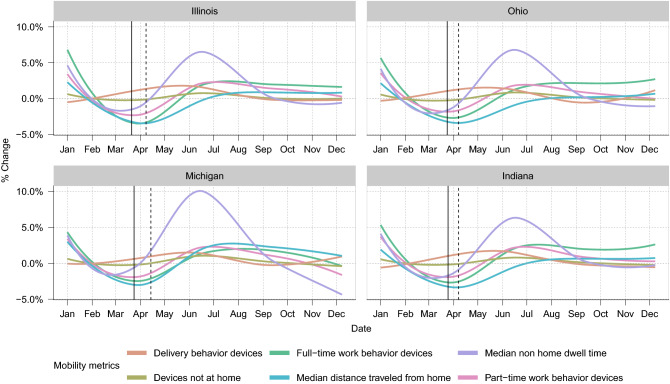
The average daily changes from baseline in the six mobility metrics for all counties of each state between January and December 2020. The baseline was calculated using a rolling average of the 7 previous days. The solid vertical lines represent the date the stay-at-home orders were put in place while the dotted vertical lines represent the dates the stay-at-home orders were lifted.

Figure 1 illustrates the average daily changes of the six mobility metrics between January and December 2020 of each state (average of all counties) relative to the week before. Overall, each metric had a unique trajectory but trends were similar across four states. Based on the average change, the number of devices not at home and delivery behavior (more than 3 stops lasting for less than 20 mins) remained stable throughout time. There was more variation in the metrics related to full-time work and the time spent away from home. Of the four states, mobility changes were more pronounced in Ohio. Across all states, relative to the previous seven days, mobility increased daily between March and May. Mobility metrics varied considerably by counties ( S1, S2, S3, S4), illustrating how aggregating changes at the state-level may mask granular changes at the county-level.

### First fPCA summarizes mobility patterns by counties

We created a latent index of mobility by counties as given by Eq. (2) which is derived from the first fPCA. Table 2 provides the median and inter quartile range of the proportion of variance explained by the first fPCA across counties in a given state. We saw that more than 50% of the variance was explained by the first fPCA for a majority of all the counties analyzed, indicating good dimension reduction. In Supplemental Figures S5, S6, S7 and S8, we provide the absolute Pearson correlations between our MI and each individual metric by county for Illinois, Ohio, Michigan and Indiana, respectively. Furthermore, the correlations were particularly strong with full-/part-time work behavior as well as time spent away from home. Importantly, there was significant heterogeneity across counties, which would otherwise be missed when aggregating mobility metrics at the state level.

**Table 2:** Median (inter quartile range) of the proportion of variance explained by the first fPCA by state. *n* represents the number of counties in each state.

Illinois (*n* = 102)	Ohio (*n* = 88)	Michigan (*n* = 83)	Indiana (*n* = 92)
0.57 (0.50, 0.63)	0.71 (0.67, 0.74)	0.61 (0.53, 0.69)	0.65 (0.59, 0.70)

**Figure 2:**
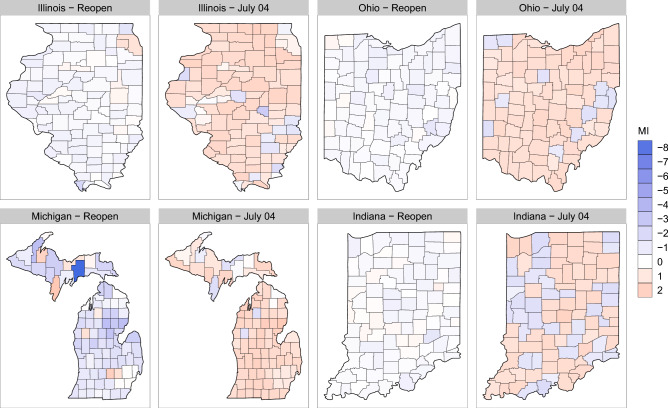
MI values for each county of each state on the day the stay-at-home orders expired (reopen) and on July 4, 2020. Blue shades indicate a decrease in mobility (MI < 0) and red shades indicate an increase in mobility (MI > 0).

Figure 2 compares the changes of the MI from the day stay-at-home policies expired and July 4 (Independence Day). Blue shades indicate MI <0 (decrease in mobility) and red shades indicate MI>0 (increase in mobility). This graph provides some evidence that our MI is appropriately capturing mobility as we would expect there to be more movement on a traditionally busy U.S. holiday compared to earlier on in the pandemic when stay-at-home orders were lifted. In the Supplemental material, we also provide an animation illustrating the daily changes from reopening to December 8, 2020. The animation shows substantial difference in mobility patterns across counties that vary from day to day. The most dramatic change over time is increases in mobility from a weekday to a weekend.

### Association with COVID-19

**Figure 3:**
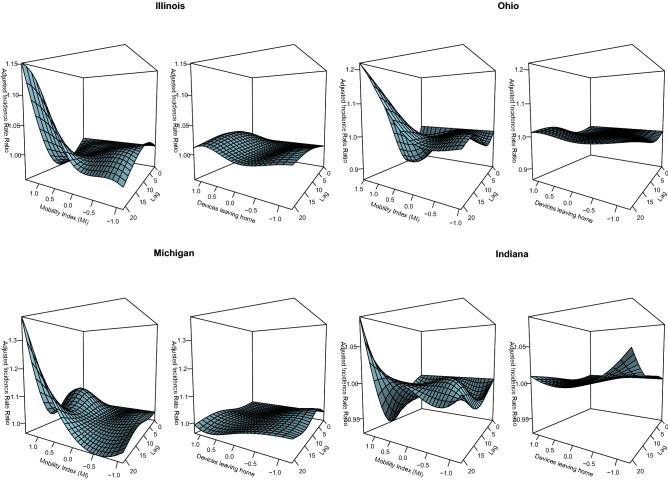
Model results comparing the MI and its association with COVID-19 cases and a commonly used single metric of mobility (fraction of devices leaving home). For each state, the left panel summarizes the multidimensional MI; the right panel represents the percentage of devices leaving their home (x-axis); y-axis is the adjusted incidence rate ratio of COVID-19, at varying lagged response (0–21 days) (z-axis).

To evaluate the validity of the MI, we compared its association with COVID-19 cases and a commonly used single metric of mobility (fraction of devices leaving home) (Fig. 3). Notably, the single metric was not associated with COVID-19 cases in any state at any lagged time point. However, there was a clear dose-response relationship between our MI and COVID-19 cases following a 10–21-day time lag in all four states. Across all four states the MI model resulted in significantly better goodness-of-fit statistics compared to the single metric (Table 3).

**Table 3:** Analysis of deviance table comparing the goodness of fit between the MI model (fPCA) and the fraction of devices leaving home (single) of the four states. Degrees of freedom shown is for the $\chi ^2$ test statistic.

State	Residual deviance	Degrees of freedom	Reduction in deviance for the MI model (fPCA)
Illinois (single)	152,581		
Illinois (fPCA)	149,282	3.6	3,299
Ohio (single)	123,551		
Ohio (fPCA)	112,975	8.6	10,576
Michigan (single)	261,759		
Michigan (fPCA)	250,888	7.0	10,871
Indiana (single)	83,517		
Indiana (fPCA)	80,297	1.7	3,220

## Discussion

The COVID-19 pandemic is now fueled by highly transmissible variants of concern. Understanding the association between mobility and disease transmission can help tailor non-pharmaceutical interventions to mitigate outbreaks and potentially be used as an early indicator for surges in new infections. We leveraged freely available cell phone data with an unsupervised machine learning approach to create a multidimensional index of mobility. Results from our study suggest following the expiration of stay-at-home physical distancing policies, single metrics of mobility were not sensitive enough to capture the complexity of human mobility related to disease transmission. Our MI was correlated with COVID-19 cases from all counties in Illinois, Ohio, Michigan and Indiana. In comparison, the single metric of mobility (fraction of devices leaving home) was not associated with incident cases. Our results also demonstrate the importance of evaluating changes at a granular level as there was significant heterogeneity within states.

Tracking mobility has the potential of becoming a powerful tool to determine the impact of public health policies^25^. A growing subfield of COVID-19 research involves the analysis of mobility data and patterns. In the last 2 years, a variety of metrics and sources have been used to track mobility^49,50^. Initial studies evaluated how populations adhered to stay-at-home policies by tracking mobility trends^11,51^. Later it became evident that mobility may be useful as a public health surveillance tool as studies evaluated the correlation between mobility and COVID-19 diagnoses.^3,52–59^ A study by Lasry et al. used Safegraph mobility data as a proxy for social distancing in the metropolitan areas of Seattle, San Francisco, New York City, and New Orleans and found an association between changes in mobility (% personal mobile devices leaving home) at the state-level and COVID-19 cases during the first COVID-19 wave^9^. In all four metropolitan areas, the number of mobile devices leaving home declined from 80% (on Febraury 26 2020) to 42% in New York City, 47% in San Francisco, 52% in Seattle, and 61% in New Orleans as stay-at-home policies were implemented. However, at this time NPI were more homogenous across counties and states. In contrast our study evaluated the association of mobility and COVID-19 cases following the expiration of stay-at-home orders reflecting mobility behavior that is more reflective of typical population-level movement. While we have used mobility data from Safegraph, several other mobility datasets have have been studied with regards to COVID-19. Zachreson et al. used data from Facebook to determine the validity of aggregate human mobility data for COVID transmission patterns^60^ . Aggregate mobility data is available through the Facebook Data For Good Program, which uses the mobile apps’ location service records of the users GPS locations. To be captured in this database, participants must be a member of Facebook and enable location services. To prevent users from being identified, Facebook removes users who do not meet a certain threshold during the data aggregation period. This means that less densely populated areas are most likely to be underrepresented by Facebook mobility data. Google also provides another source of aggregate mobility using the time spent by users at several geolocations using Google Maps. While studies using Google data have found that increases in mobility lead to increased COVID-19 cases and death^61^ , to date studies have not taken into account changes in mobility following the end of stay-at-home policies. Apple Mobility also provides mobility data using Apple Maps to create aggregated counts of direction requests. A study by James and Menzies used Apple Data and found mobility data and national financial indices exhibited similarities in their trajectories. Apple Mobility has several limitations as it includes any searches for directions as a measure of mobility and therefore may not be representative of actual community mobility.^62^

Mobility data offers several functions as a public health tool. While we focused directly on the number of COVID-19, cases, mobility can also be used to estimate and model of transmission rates^52,60^. Spatially explicit models of disease transmission using census data are often used to guide disease intervention decisions. However, it remains important to define mobility as a multidimensional construct. We demonstrated among hundreds of counties from four states, time-updated relative changes were associated with increases in COVID-19 cases. Furthermore, results from our study suggest our mobility should be considered an important confounder when evaluating the impact of other non-pharmaceutical interventions.

The strength of our study was the use of multiple advanced statistical methods to measure mobility and then validating its utility by evaluating its association with COVID-19 cases. The fPCA used to create the mobility index effectively captured the heterogeneity of the individual metrics over time and across counties within a given state. The unsupervised nature of this approach prevented the model from overfitting when evaluating the association with cases. Furthermore, we modelled a non-linear functional relationship between mobility and COVID-19 cases using a HGAM model while simultaneously fitting different lagged time periods. The expectation that the lag time should vary across states was confirmed by our results. The use of these methods has been under appreciated in the epidemiological and public health studies; we provide code and data to expand the use as we believe these methods could have wide applications in future research. We also highlight the need to track population-level mobility at a granular level, as we show significant heterogeneity across counties.

Our study also has limitations. The results of our study are based on data from all 365 counties from four states in the Midwest. While these counties represented varying population densities, socioeconomic conditions, and party affiliations (that may have resulted in different adherence and uptake of other NPI) our results may have limited generalizability to other larger metropolitan cities. Cell phone data was freely available and could help to predict trends during the pandemic but it is only a proxy for human contact. In this study we attempted to define a more robust definition of mobility, however it still remains a surrogate exposure. The association between mobility and COVID-19 cases may be underestimated, given our outcome is dependent on testing. Testing capacity has significantly changed throughout the pandemic in the United States. Seroprevalence studies estimate case detection is underrepresented by a factor of three times^63^. Although we do not believe this underrepresentation is differential between counties, outcomes such as COVID-19 related deaths and hospitalizations may be less biased. While the advantage is clear, the utility of these outcomes as a “real-time” public health tool is debatable as the latency period (time of infection to outcome) is long (greater than 21 days). As with all observational studies, associations should not be interpreted causally. Our model does not take into consideration confounding interventions that could also increase or mitigate transmission such as the proportion of the population adhering to physical distancing guidelines, wearing masks, interactions outside vs inside or air quality. To effectively measure social distancing patterns using individual-level data (either cell phones or wearable technology such as fitness trackers), would be more sensitive compared to aggregate data, but this raises ethical and privacy concerns^64^. Recent reports have hypothesized the COVID-19 pandemic may not be following a normal distribution but over dispersed or driven by “super spreader” transmission events which we did not account for in our model^65^. Finally, while PCA has the advantage of reducing overfitting, it has several assumptions and limitations. We must assume the features are related to each other in a linear fashion, and that the data can be appropriately summarised by the mean and variance^66^. Furthermore, PCA can be heavily influenced by outliers (three times the standard deviation from the sample mean), requires that the PCs are orthogonal to each other, and results in information loss due to selecting a relatively small number of PCs for downstream analysis. Specifically for our data, we show in Table 2 that selecting the first fPCA explains over 50% of the variance explained for a majority of all the counties analysed. The data did not have any significant outliers as seen in Supplemental Figure S13, which shows that the coefficients of variation (standard deviation divided by the mean) is less than 2.5 for all mobility metrics across all counties. We did not pursue nonlinear dimension reduction techniques such as kernel PCA, but think this would be an interesting direction for future research.

## Conclusion

Our study underscores the potential of using freely available cell phone data as public health tool. We show changes in mobility can be used a predictor of surges in COVID-19 cases. However, monitoring mobility in the absence of strict non-pharmaceutical interventions such as stay-at-home policies will require robust definitions.

## Supplementary Information

Supplementary Information.
