# Supplementary material for: Novel mobility index tracks COVID-19 transmission following stay-at-home orders
Source: Sci Rep. 2022 May 10;12:7654. doi: 10.1038/s41598-022-10941-2 (PMC9088135; doi:10.1038/s41598-022-10941-2)
Supplement: Supplementary file 1 — Supplementary Information. [file 41598_2022_10941_MOESM1_ESM.pdf]

Novel mobility index tracks COVID-19 transmission following stay-at-home orders

Peter Her[1,2,+], **Sahar Saeed**[3,+], **Khai Hoan Tram**[4], **and Sahir R Bhatnagar**[5,6,*]

[1]Department of Pharmacology and Therapeutics, McGill University
[2]Department of Medical Biophysics, University of Toronto
[3]Division of Infectious Diseases, Department of Medicine, Washington University School of Medicine
[4]Division of Infectious Diseases, Department of Medicine, University of Washington, Seattle
[5]Department of Epidemiology, Biostatistics and Occupational Health, McGill University
[6]Department of Diagnostic Radiology, McGill University
[*]sahir.bhatnagar@mcgill.ca
[+]these authors contributed equally to this work

A County-level mobility metrics

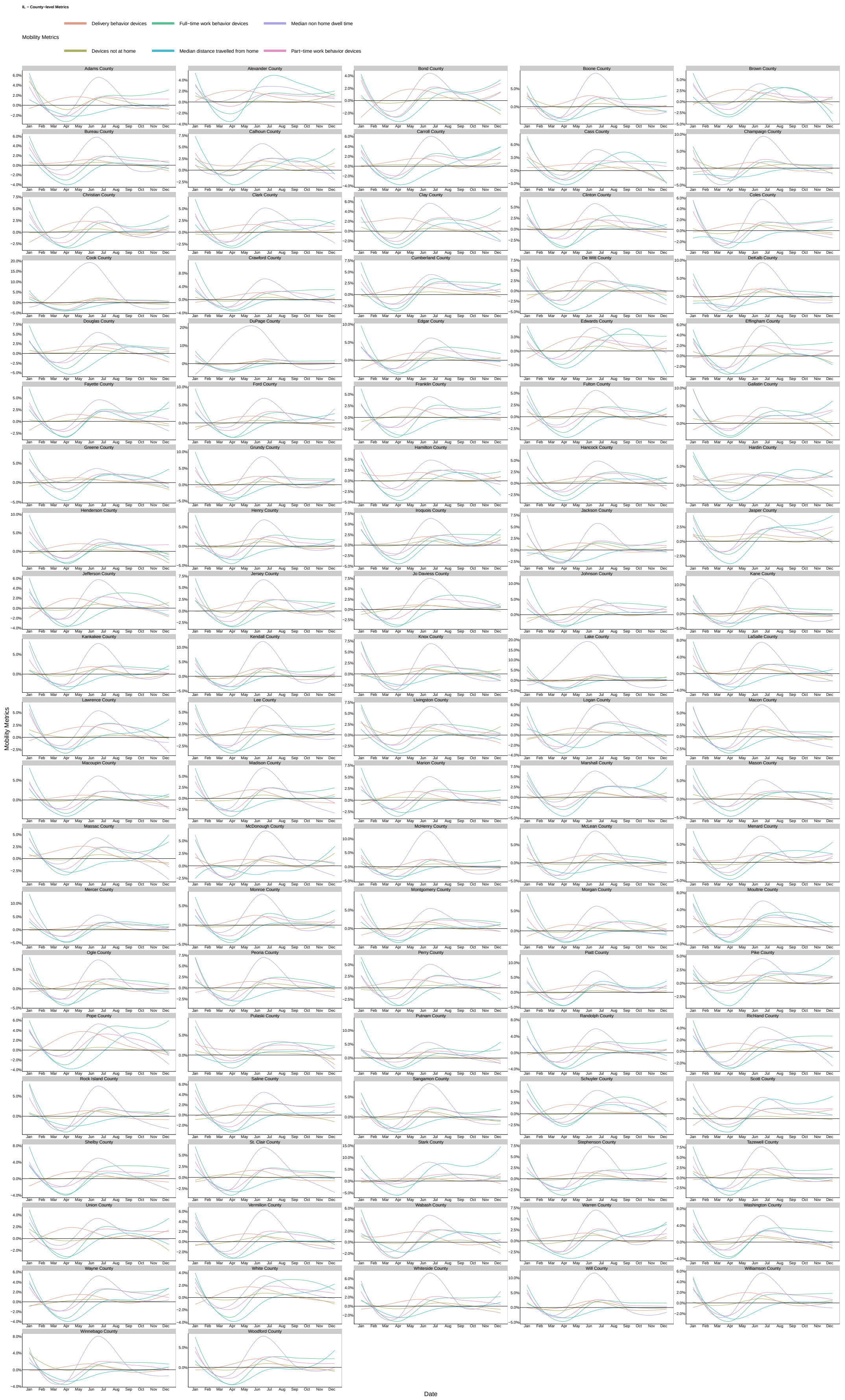

Figure S1. Illinois

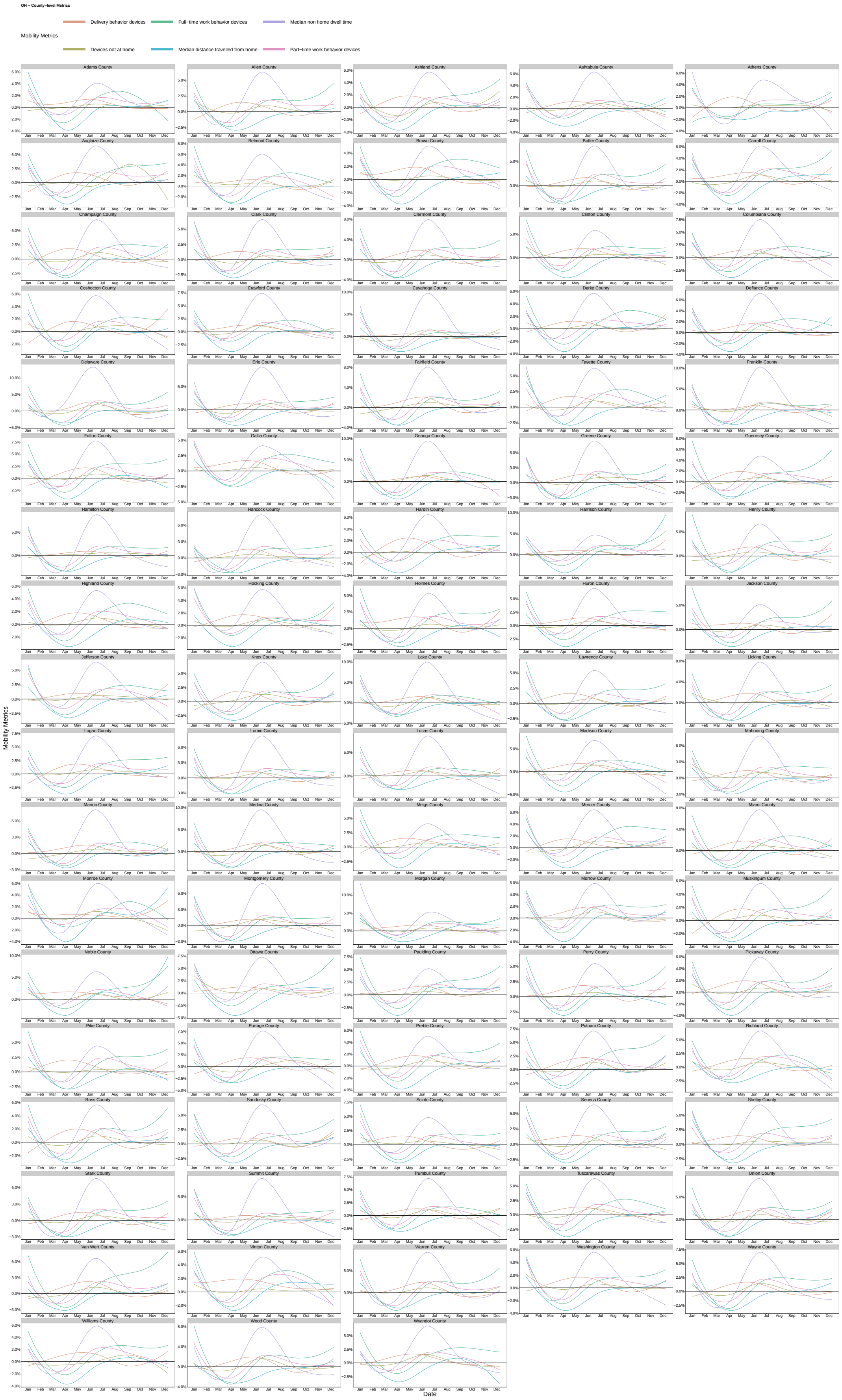

Figure S2. Ohio

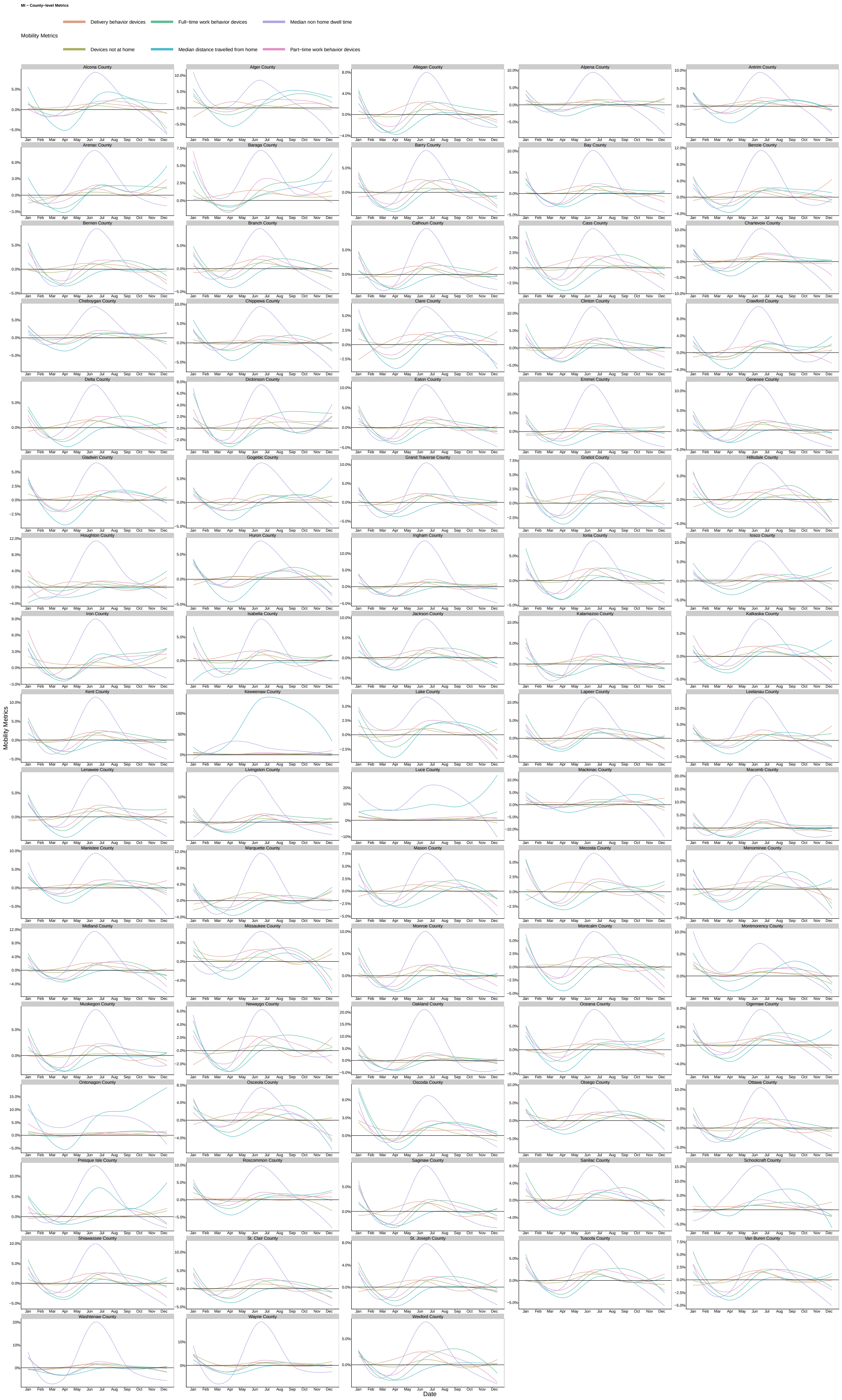

Figure S3. Michigan

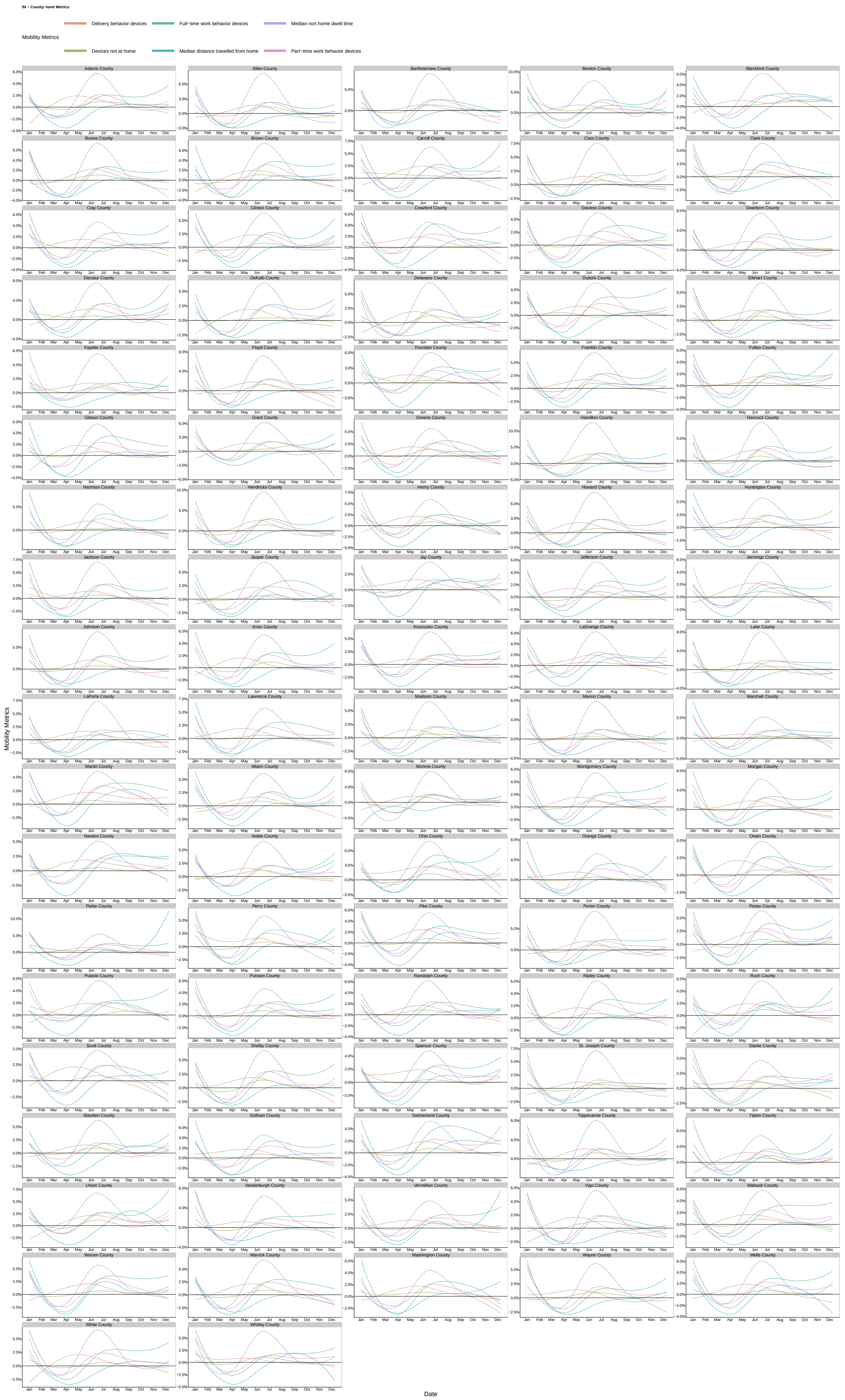

Figure S4. Indiana

B Pearson correlations between MI and individual metrics

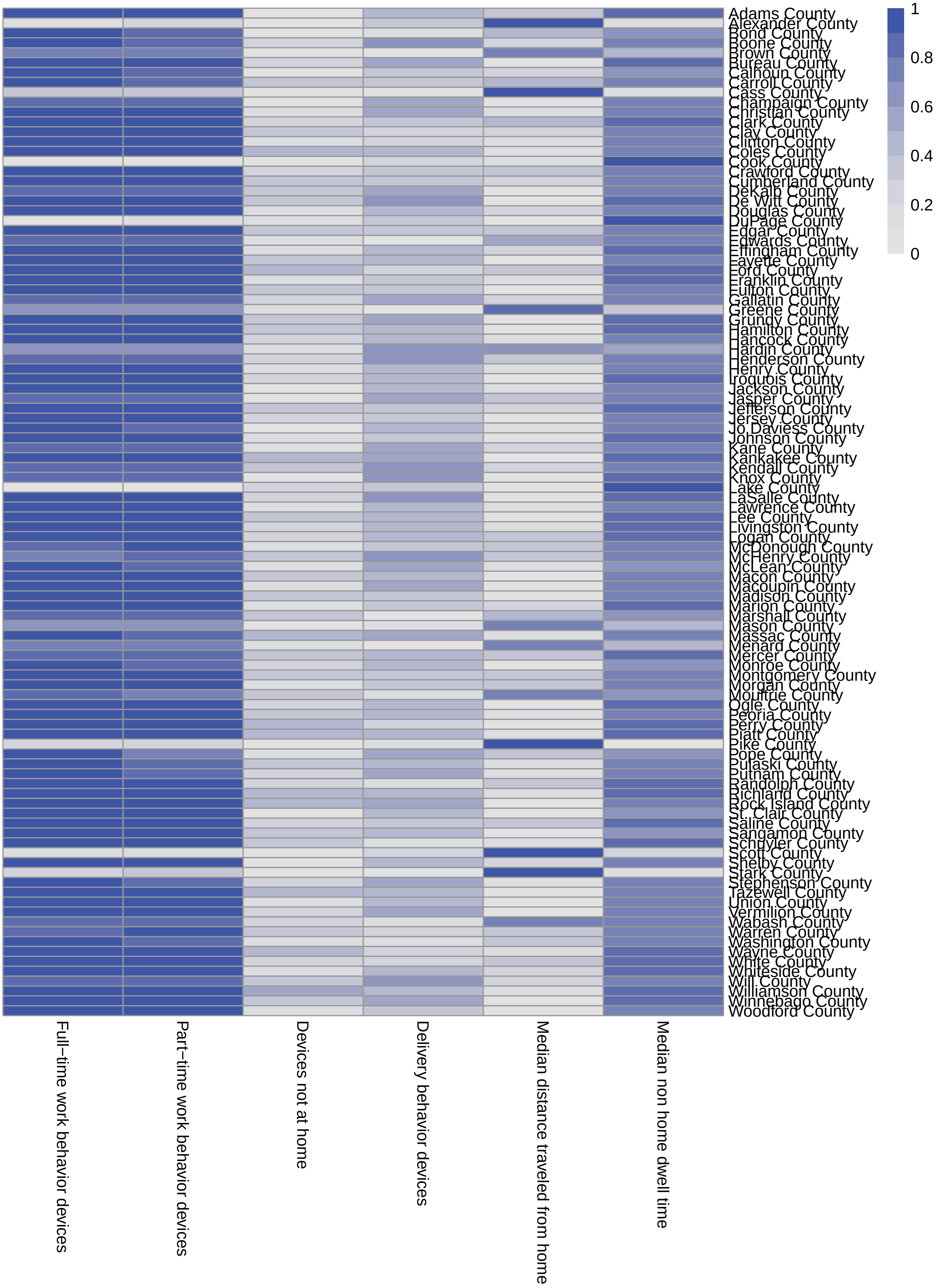

Figure S5. Illinois

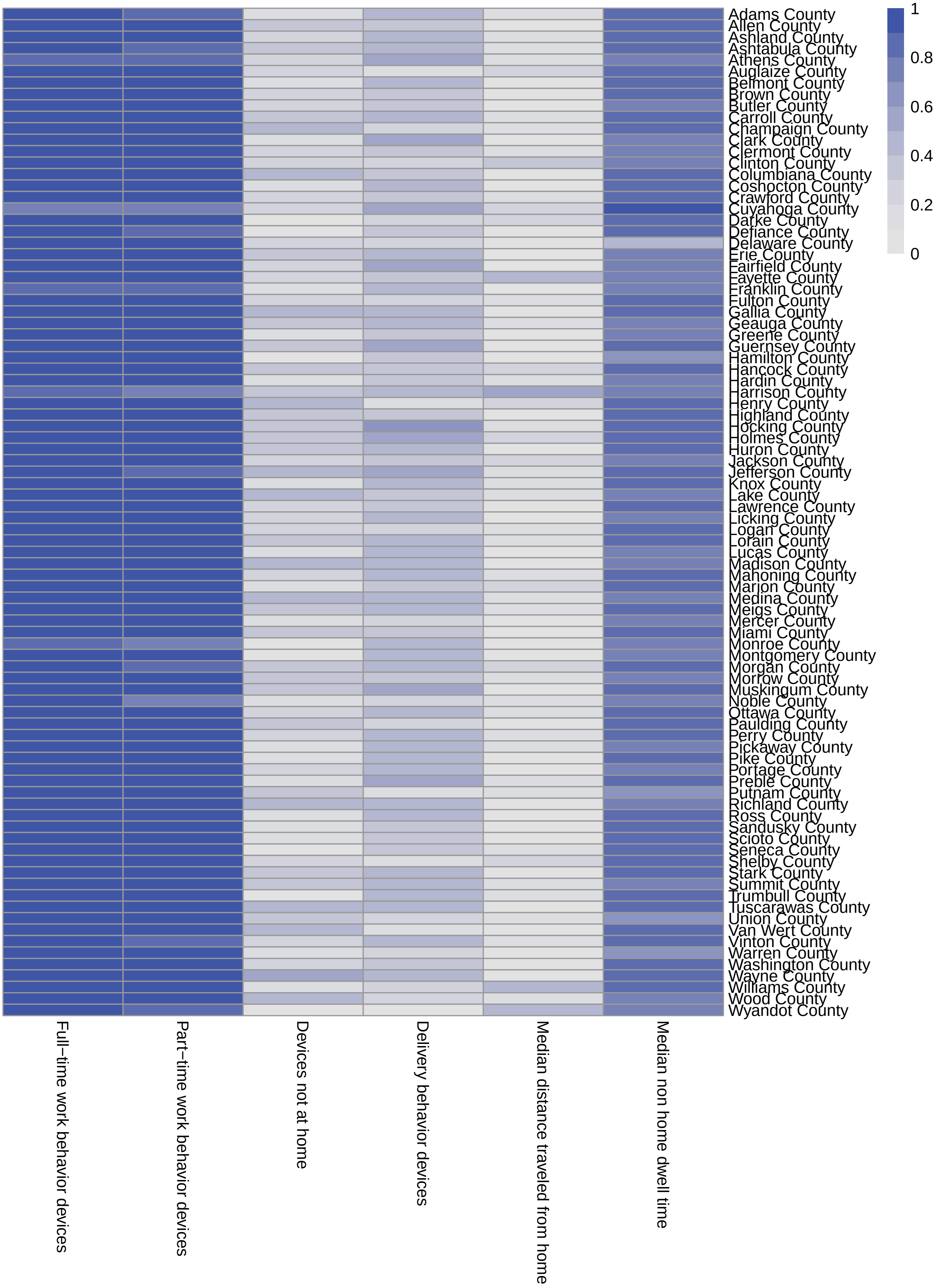

Figure S6. Ohio

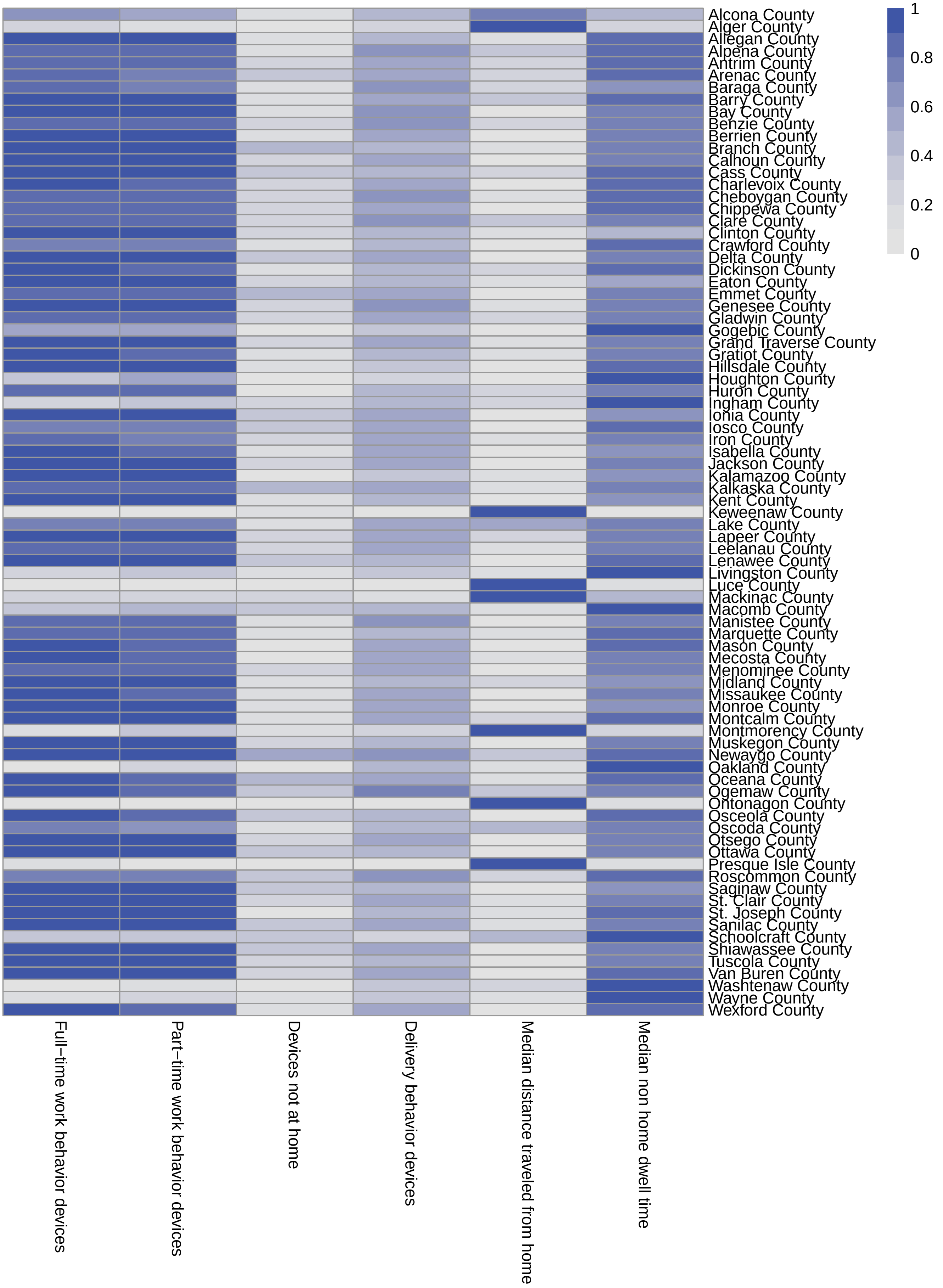

Figure S7. Michigan

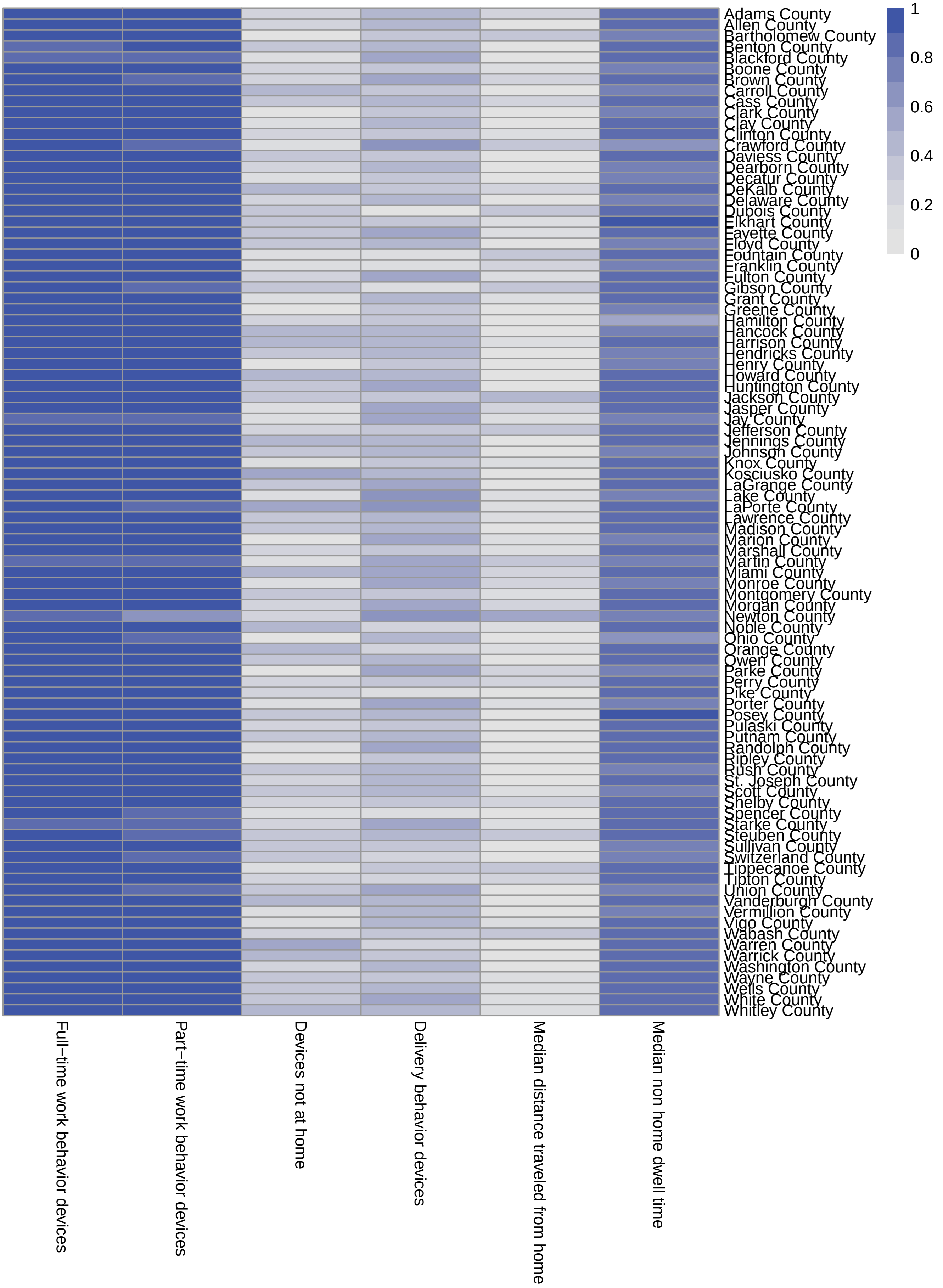

Figure S8. Indiana

B.1 p-values of correlations between MI and individual metrics

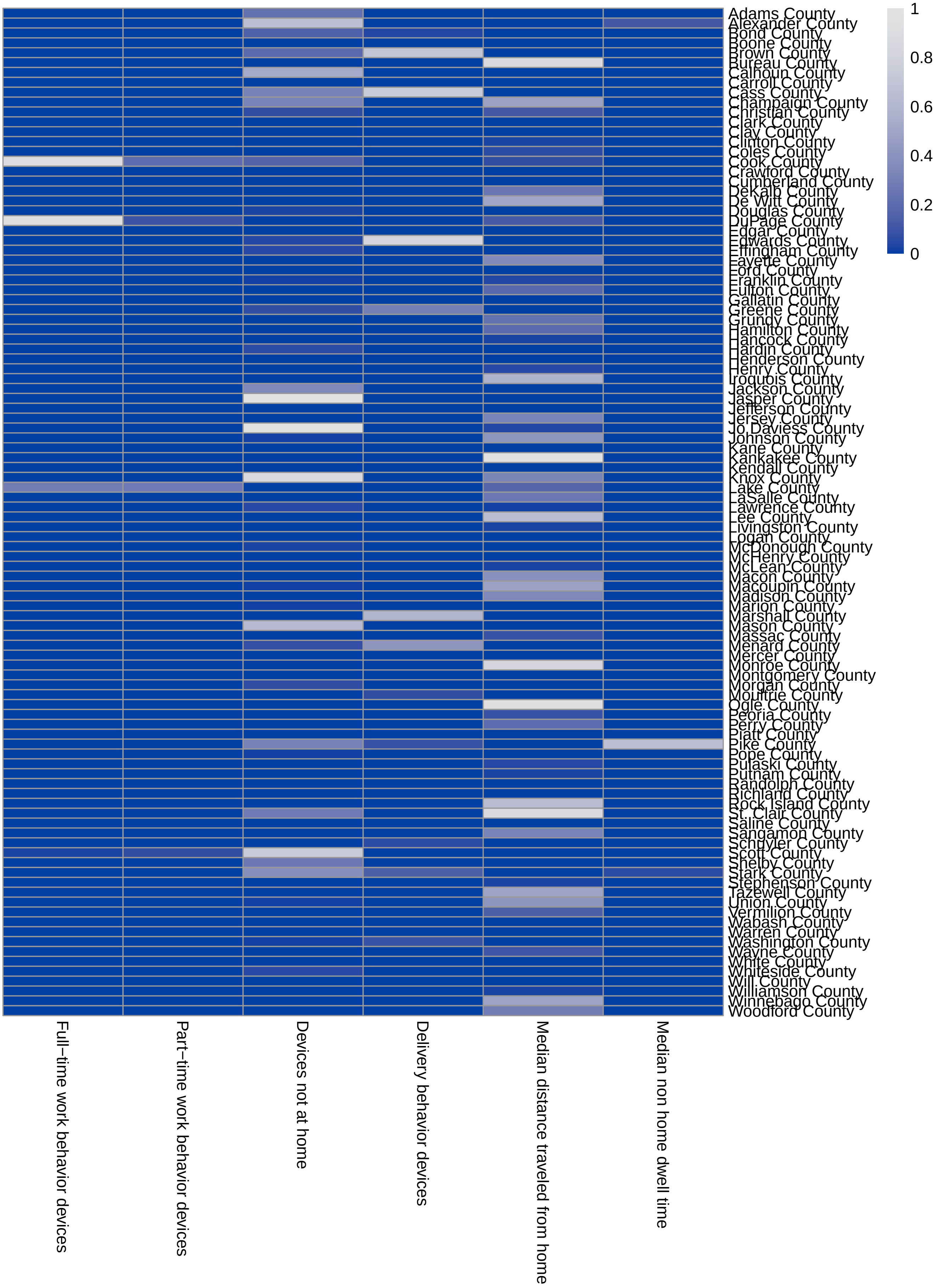

Figure S9. Illinois

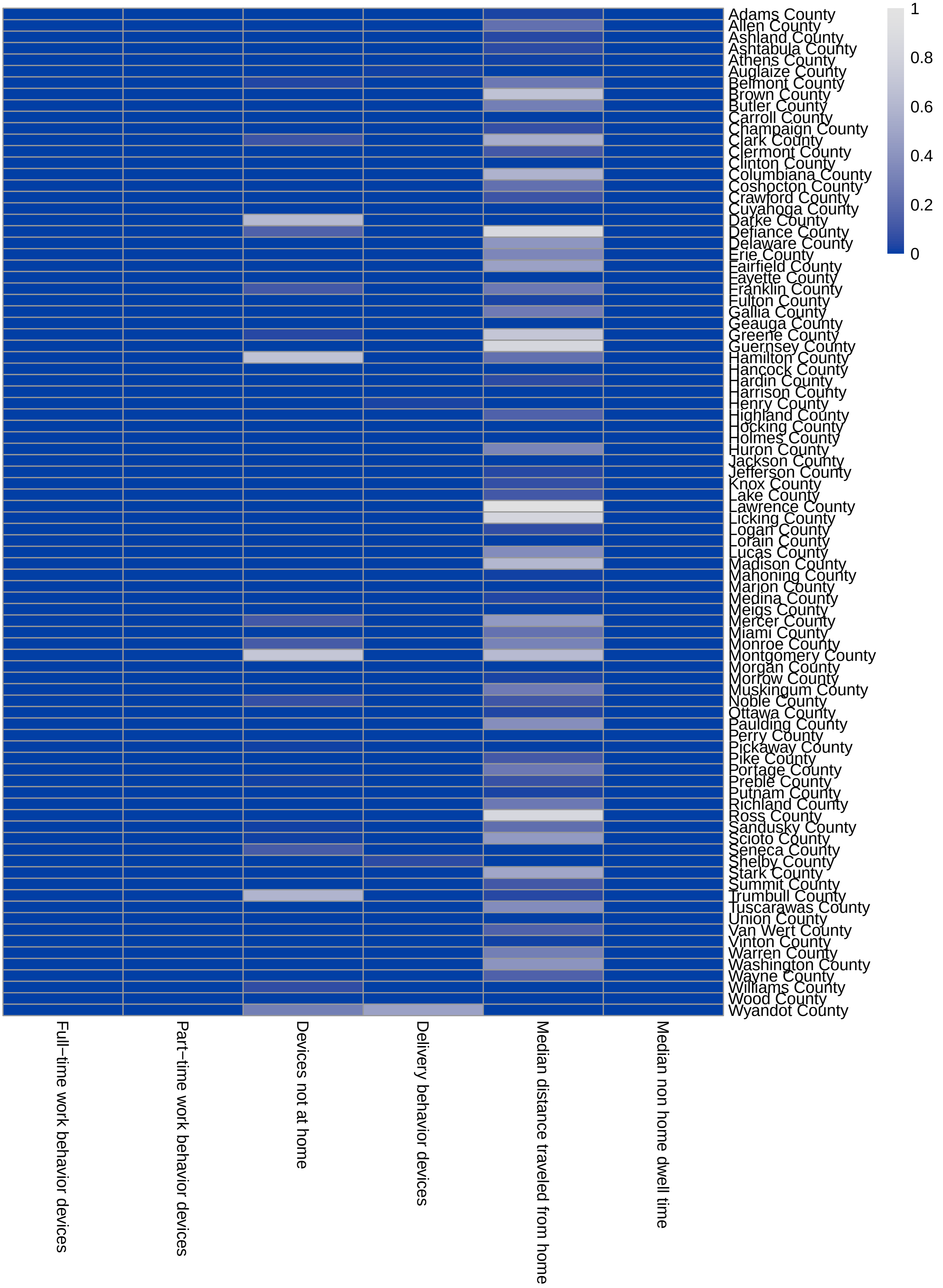

Figure S10. Ohio

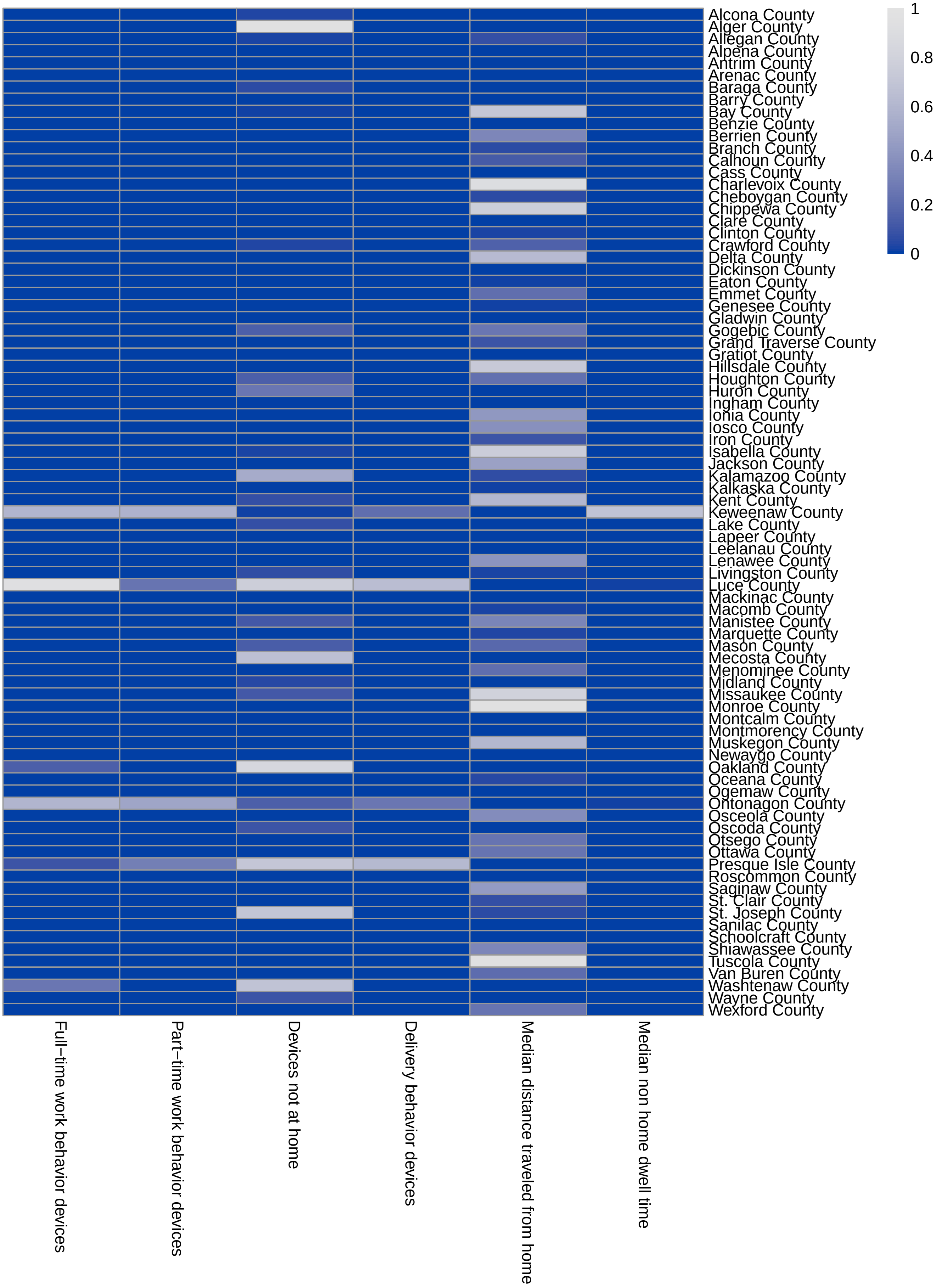

Figure S11. Michigan

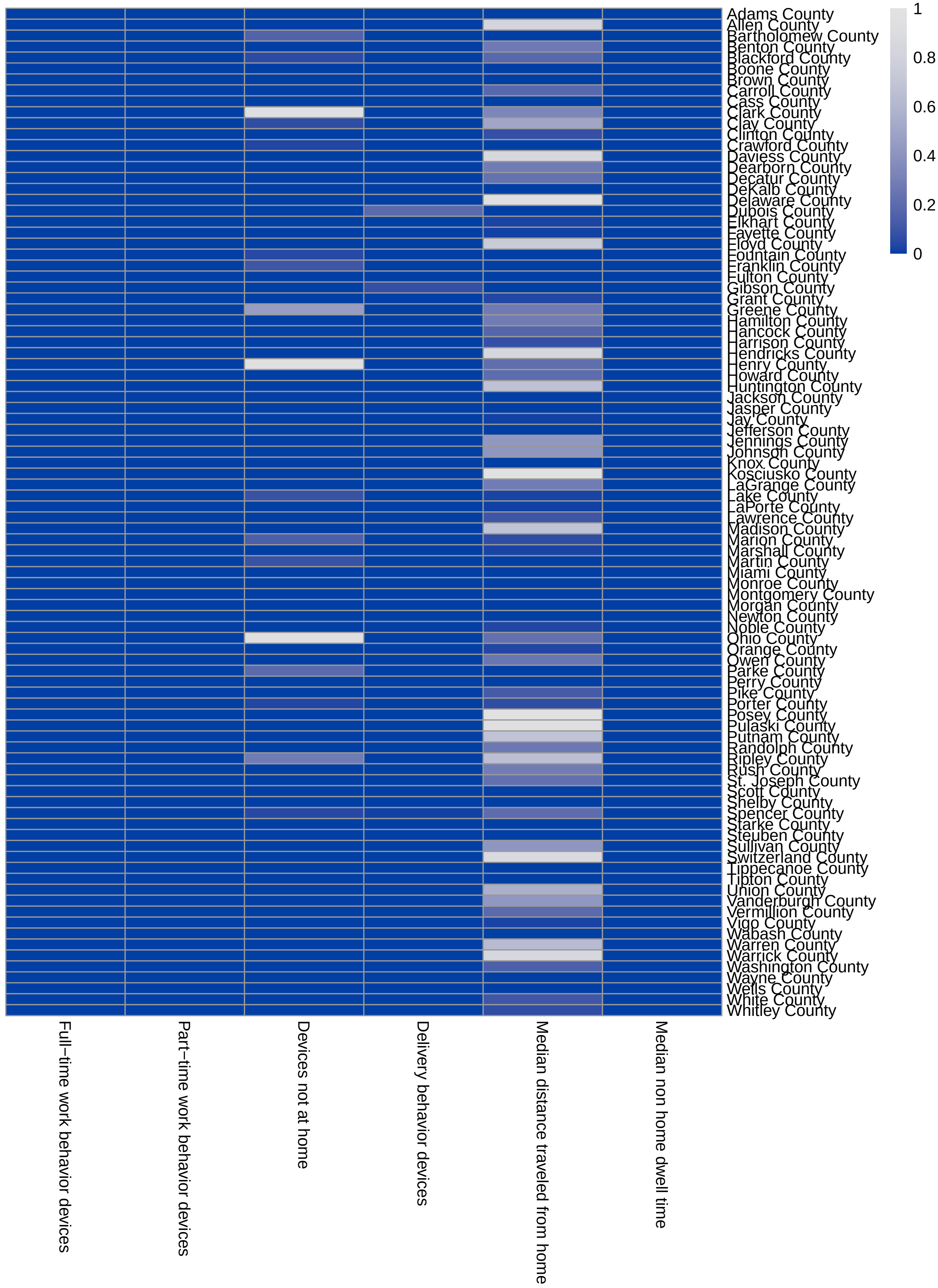

Figure S12. Indiana

C Coefficient of variation for each individual metric

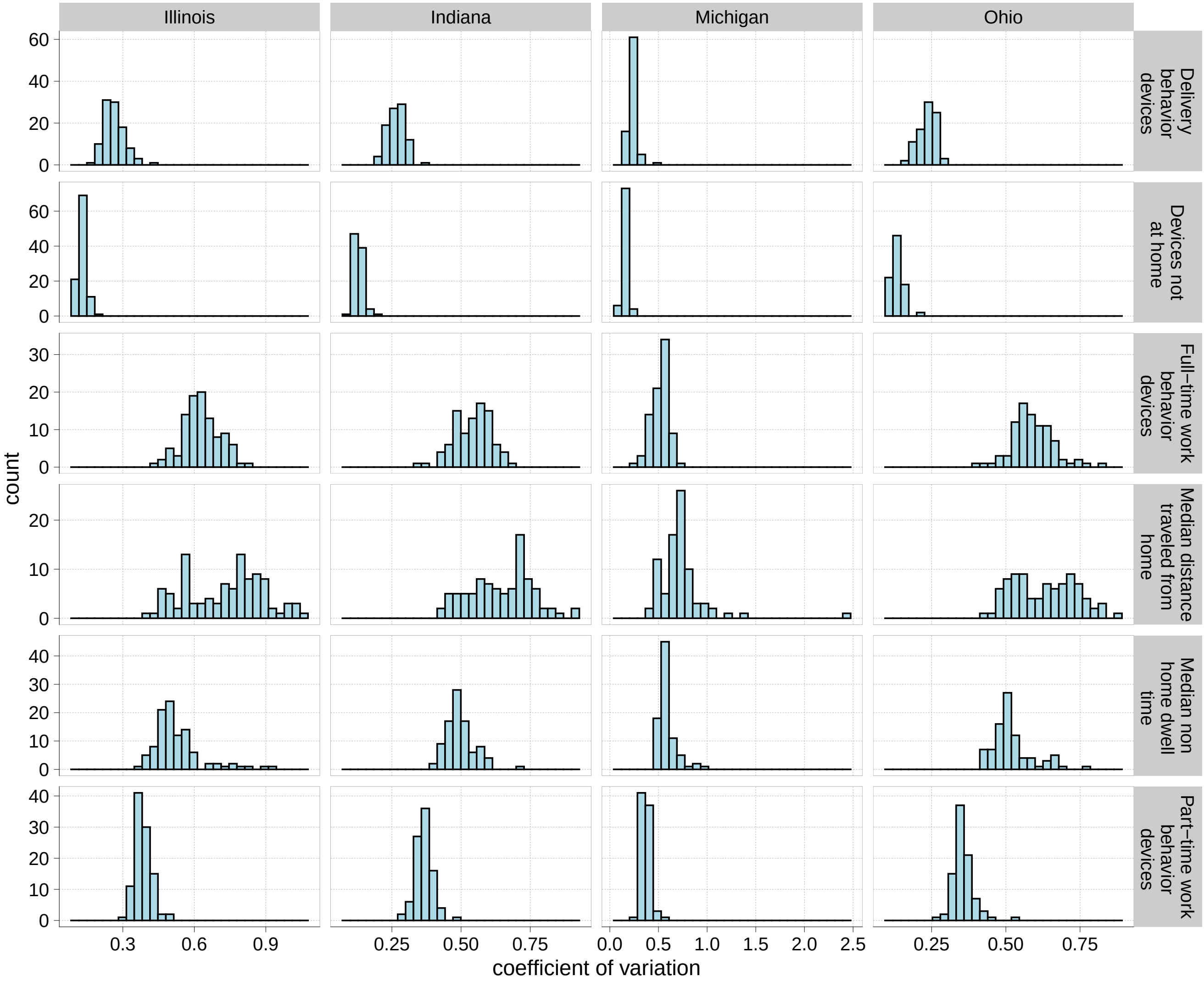

Figure S13. Coefficient of variation (CV) for each individual metric and county. The histograms in each state represent the distribution of CV for each county and mobility metric.
